# Interspecific patterns of small cats in an intraguild-killer free area of the threatened Caatinga drylands, Brazil

**DOI:** 10.1371/journal.pone.0284850

**Published:** 2023-04-21

**Authors:** Lester Alexander Fox-Rosales, Tadeu Gomes de Oliveira

**Affiliations:** 1 Department for the Ecology of Animal Societies, Max Planck Institute of Animal Behavior, Konstanz, Germany; 2 Department of Conservation Biology, Georg-August Universität, Göttingen, Germany; 3 Programa de Pós-Graduação em Ciência Animal da Universidade Estadual do Maranhão, São Luís, Maranhão, Brazil; 4 Departamento de Biologia, Universidade Estadual do Maranhão, São Luís, Maranhão, Brazil; Cheetah Conservation Fund, Namibia University of Science and Technology, NAMIBIA

## Abstract

The semi-arid Caatinga is the largest dry forest ecoregion in the Americas; nevertheless, it is experiencing alarming rates of habitat loss. Most vegetation fragments in the biome are either unprotected or within private lands; however, these private areas are susceptible to anthropogenic activity, and often have the presence of non-native wildlife such as domestic dogs and cats. Two small felid species, the northern tiger cat and the jaguarundi co-occur throughout the Caatinga and have overlapping niches, which require segregation mechanisms to avoid interference competition. Assessing these species strategies for coexistence is crucial, as it can guide conservation actions. With this aim, a private ranch in the Brazilian Caatinga drylands was surveyed and multi-species occupancy models were used to assess co-occurrence patterns between northern tiger cats and jaguarundis. The degree of temporal overlap between both felids and domestic dogs and cats were also assessed. Evidence was found of positive co-occurrence between tiger cats and jaguarundis, suggesting a lack of spatial segregation at our study site; and low temporal overlap was found between both felids, with tiger cats being nocturnal and jaguarundis diurnal. High temporal overlap was found though between domestic dogs and both wild felid species. Our results suggest that small felids can coexist in private areas of the Caatinga with sufficient habitat. However, there is a need to highlight the potential threat of disease transmission by non-native carnivores as something that should be addressed in these private landscapes.

## Introduction

Interspecific interactions are one of the key factors that shape species behavior and occurrence [1–4]. Species occurrence and distribution are also shaped by environmental constraints. These two forces, the environmental factors and the presence or absence of other species, heavily influence where a species occurs. For example, in a community containing two competing species, the subordinate competitor may forgo areas of good habitat quality because of the presence of the dominant competitor in such areas [5–7]. This also applies to the presence of interspecific killers, which, in the case of the Neotropics, tend to be the large-sized jaguar (*Panthera onca*) and puma (*Puma concolor*) and the medium-sized ocelot (*Leopardus pardalis*) [8]. In the small Neotropical felid guild [9], it is the dominant mesopredator, the ocelot, not the larger cats, that negatively affects the density of the smaller jaguarundi (*Herpailurus yagouaroundi*), margay (*Leopardus wiedii*), northern tiger cat (*Leopardus tigrinus*), and southern tiger cat (*Leopardus guttulus*) [10,11]. These smaller felids co-occur with the larger ocelot throughout the Neotropics, and in most areas they exhibit low density and relative abundance [9,12]. Their long-term conservation has been suggested to be highly dependent on smaller private lands, where ocelots are rare or absent [9,13–15]. In the Argentinean Atlantic Forest for example, ocelots are abundant in well-protected sites, whereas margays and southern tiger cats use the disturbed sites more, where poaching and selective logging take place [16]. Thus, understanding the factors that influence small felid habitat use in private areas, is key for effective conservation and management actions.

In order to properly assess the occurrence patterns of small felids, it is necessary to take into account both environmental factors and interspecific interactions. Though the negative influence of ocelots on smaller felids is better documented [9,12], less research has focused on the interactions between the smaller felids themselves in areas where ocelots are absent. Moderate to high levels of dietary and spatial overlap among small Neotropical felids have been documented at some study sites [9,17,18]. Species with overlapping diets and space use may shift their activity patterns to avoid antagonistic encounters with potential competitors [19–21]. Thus, segregation among the temporal niche dimension could be an important coexistence mechanism for small Neotropical felids. Testing whether the potential for direct encounters has a marked effect on small felid habitat preferences is necessary, as data on spatial and temporal ecology is very relevant for conservation purposes. Furthermore, within privately owned areas there are other threats and factors that could influence the long-term conservation outlook of small felid species. These areas are often located next to or within agricultural and human-dominated landscapes, where the risk of poaching is higher [22]. Additionally, the presence of domestic animals, such as dogs and cats, may pose a threat (i.e. predation and/or disease transmission) in some areas [23–26]. All these factors must be taken into account when modeling the spatial ecology of small felids in private lands.

The jaguarundi is a small felid (roughly 5.2 kg), largely terrestrial and diurnal [27,28]. The northern tiger cat is even smaller (roughly 2.4 kg), and it is a globally threatened species; classified as Vulnerable by the International Union of Conservation of Nature [15,29]. Both species are threatened in Brazil, the former is classified as Vulnerable and the later as Endangered [30,31]. Their respective ranges overlap extensively, hence conservation actions targeting both species simultaneously in private lands could be possible provided some form of ecological segregation allows them to coexist, even in marginal habitats. To find out the mechanism of coexistence between these two species, their habitat use patterns were analyzed in a small private reserve of the semi-arid Caatinga, a biome composed of thorny scrub and tropical dry forests located in northeastern Brazil. Since none of their potential interspecific killers are present in the area, they should not pose any limitations for either of the small cats. Environmental preferences in a multi-species occupancy framework were modeled considering the interaction between both species. This modeling approach not only allows us to estimate habitat selection preferences for each species, but also whether habitat selection differs in the presence or absence of the other species. Temporal overlap between both felids and domestic dogs and cats, which are present at our study site, were also explored to quantify the degree of potential threat from the two domestic species. As such, the following research questions were addressed: Is there evidence of co-occurrence affecting habitat use patterns of both felids? Is there temporal segregation between jaguarundis and tiger cats? What is the degree of temporal overlap between both wild felids with respect to domestic dogs and cats? The following hypotheses were tested: 1) Tiger cats will prefer denser areas [32,33], while jaguarundis will use both open and dense formations [34–36]; 2) Temporal-mediated segregation between both wild felids [28,37]; 3) High temporal overlap between wild felids and domestic carnivores [38].

## Materials and methods

### Study site

**Fig 1:**
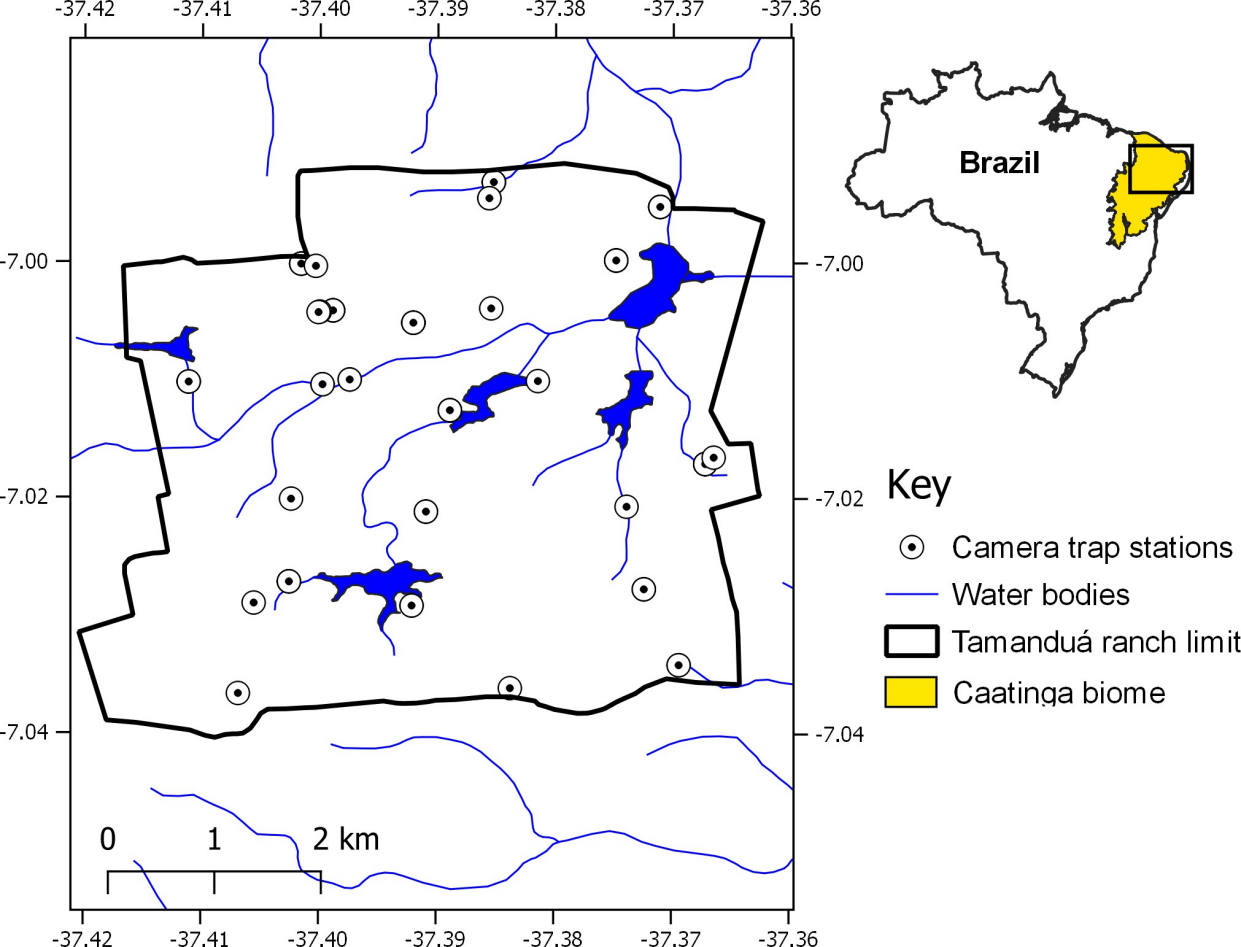
Study area and camera trap stations at the Tamanduá Ranch in the Brazilian Caatinga.

The Tamanduá Ranch is a 3,073 ha private ranch located in the state of Paraíba, northeastern Brazil (Fig 1). Roughly one third of the property is protected and preserves a range of native Caatinga vegetation, including open grasslands, shrublands, thorny scrub, and dense dry forests with higher canopy cover. All permanent water sources in the area are man-made, and riparian vegetation flourishes among some of these water bodies. The non-protected area is used for dairy farming, small-scale organic agriculture, beekeeping and fruit harvesting. The climate is hot and semi-arid (type BSh), with a mean annual rainfall of 700 mm (falling mostly between January and April), mean annual temperatures of 20.8–32.9°C [39] and an elevational gradient from 200–400 m above sea level.

The area contains several mammalian species that are typical of the Caatinga biome [39], but it has lost its main apex predator, the puma (*Puma concolor*) as well as the ocelot (*Leopardus pardalis*). After monitoring the area with camera traps between 2010–2014 and then again between 2018–2022 (combined total sampling effort 24,831 trap-nights), not a single record of either species has been obtained [32], suggesting they are likely extirpated. Therefore, our study site offers us a unique opportunity to analyse segregation mechanisms among small felids in the absence of larger competitors.

### Camera trapping

A total of 18–24 camera trap stations were deployed across a grid (30 cells of 1 km x 1 km each) from August 2018 through June 2021. Grid cells were laid out in areas of Caatinga vegetation with varying degrees of tree cover (open scrubs, shrublands, and denser dry forests). Camera placement within a grid was based on accessibility, with a minimum spacing of 500 m between adjacent camera traps, and based on the minimum distance traveled by small Neotropical felids according to telemetry studies [28,40,41]. At each trapping station, a single non-baited camera of the following brands was placed: Reconyx PC850 (Reconyx Inc., Holmen, Wisconsin), ScoutGuard SG565 (Boly Inc., Santa Clara, California), or Browning Dark OPS Pro XD (BTC, Birmingham, Alabama). Cameras were attached to trees or stakes at a height of 30–50 cm above the ground. All camera stations were located on trails and natural pathways in order to maximize detection of felids and other wildlife. Because camera traps are a non-invasive tool with no animal captures involved, this study did not require clearance from an animal ethics board.

Camera trap images were analyzed using the ‘camtrapR’ package [42]. Independent records of a given species were defined as consecutive photos more than 60 minutes apart for the same individual and photos of the same species but different individuals at any time interval [43].

### Environmental variables

For the study, three spatial covariates were measured since it was hypothesized that they likely influence small felid habitat use patterns (Table 1). These covariates are distance to permanent water sources, distance to nearest plantation, and percent tree cover. The environment at our study site is semi-arid, hence water should be a limiting factor for wildlife. Plantations at the site could provide prey for both species in the form of rodents and birds [44]. Tree cover is a measure of canopy density, all camera trap stations were located in areas of native Caatinga vegetation ranging from shrublands to true tropical dry forests. It was expected that northern tiger cats would favor areas of higher canopy cover as it has been the case at other Caatinga sites [32,33]. On the other hand, jaguarundis are known to use a wide variety of vegetation types, so a significant response to this covariate was not expected [34,35]. Satellite imagery was used to digitalize water sources using QGIS ver. 3.8 [45], while percent tree cover was obtained from a published dataset [46]. Distance was measured to the nearest water source and to the nearest plantation relative to the precise camera trap location. Percent tree cover was averaged on a 50-m buffer around each camera trap [47].

**Table 1:** Summary of covariates and their predicted effects on northern tiger cat and jaguarundi occupancy and detection probabilities at the Tamanduá Ranch.

Covariate	Mean (±SD)	Predicted effect
**Occupancy (Ψ)**		
Percent tree cover (%)	27.77 (±22.35)	Tree cover should have a positive effect on tiger cat occupancy and no effect on jaguarundi occupancy.
Distance to nearest permanent water source (m)	407.81 (±342.59)	Water is a limiting resource in semi-arid habitats; hence, it should increase occupancy.
Distance to nearest plantation (m)	906.77 (±650.97)	Plantations may provide food sources for small cats and, hence, they should increase occupancy of both species.
**Detectability (p)**		
Survey effort (days/survey occasion)	10.98 (±2.93)	Higher survey effort should increase detectability of both species.
Distance to nearest permanent water source (m)	407.81 (±342.59)	Water is a limiting resource in semi-arid habitats; hence, it should increase detectability.

### Spatial co-occurrence

Spatial co-occurrence was modeled between tiger cats and jaguarundis using multi-species occupancy models [48]. These models generalize the single-season, single-species occupancy model [49] to two or more interacting species in a multivariate Bernoulli distribution. The model contains two parameters; each of which is estimated for each species: detection probability (*p*) and occupancy probability (ψ). The first is defined as the probability of detecting a species at a site given that it is present. Ψ represents the probability of occurrence at a site; though, since our species are highly mobile, it is possible that a camera trap that failed to detect either species is contained within an individual’s home range, hence we interpret Ψ as the probability of habitat use [50,51]. The latent occupancy state of each felid species is thus modeled using the following approach:

$$Z∼MVB(\Psi_{11},\Psi_{10},\Psi_{01},\Psi_{00})$$

Where *Z* represents a two-dimensional vector of 0’s and 1’s (latent presence-absence of each species), Ψ_11_ is the probability of both species occurring at a site, Ψ_10_ is the probability that only northern tiger cat occurs at a site, Ψ_01_ is the probability that only jaguarundi occurs at a site, and Ψ_00_ is the probability of neither species occurring at a site. Thus marginal occupancy for the northern tiger cat would be *P*_(*Z* = 1)_ = Ψ_11_ + Ψ_10_, whereas for jaguarundi, it would be *P*_(*Z* = 2)_ = Ψ_11_ + Ψ_01_.

Ψ is modeled in a logistic regression framework as a function of the covariates listed above. Hence, the natural parameters for a two-species model are defined as the log-odds that a species occupies a given site:

$$f_{1}=log(\frac{\Psi_{10}}{\Psi_{00}})={\chi ’}_{\alpha }\alpha$$

$$f_{2}=log(\frac{\Psi_{01}}{\Psi_{00}})={\chi ’}_{\beta }\beta$$

$$f_{12}=log(\frac{\Psi_{11}\Psi_{00}}{\Psi_{01}\Psi_{10}})={\chi ’}_{\gamma }\gamma$$

Where *f*_*1*_ represents the natural parameter for the northern tiger cat, *f*_*2*_ represents the natural parameter for the jaguarundi, and *f*_*12*_ represents the natural parameter for both species co-occurring; and χ’_α_, χ’_β_, and χ’_γ_ are vectors of covariates and α, β, and γ are vectors of their respective slope parameters.

Unlike other multi-species occupancy approaches [52–54], this model does not make *a priori* assumptions about each species dominant or subordinate status. On the basis of body size, jaguarundis could be dominant over northern tiger cats; nevertheless, there are no documented records of jaguarundis either killing or displacing northern tiger cats [8].

Due to the length of our sampling period, and because we were interested in testing hypotheses related to habitat use and co-occurrence, the data from the 3-year period was stacked. The survey was divided period into 120-day blocks, each representing an independent survey for modeling procedures. Each survey was then divided into 10 occasions of 12 days each. Several models were constructed, each representing a biologically relevant *a priori* hypothesis. First, a null model was created, which held all parameters constant. A null model fixing the *f*_12_ parameter at 0 was also created in order to test for spatial independence between both species. Models were then produced to test the effect of year on the occupancy parameter of each species. Thereafter, models were produced to test the effects of the three spatial covariates on the marginal occupancy parameter of both species. With the best occupancy model for each species, the effects of survey effort and water distance on the detection parameter were tested. Survey effort was defined as the number of days per sampling occasion in which the camera trap was active. Finally, the effect of each spatial covariate on the co-occurrence parameter was tested, and all models that failed to converge were excluded. Any covariates whose 95% confidence interval did not overlap 0 were deemed as important environmental predictors [55] and, to avoid over-parameterization, only one covariate at a time per parameter was tested. Because the sampling was biased towards the dry season, the effects of season on the detectability or occupancy of each species was not tested.

A test was performed for multicollinearity of the covariates using Spearman correlations and did not use correlated covariates (|ρ| ≥ 0.7) in the same model. All covariates were converted into z scores to a mean of zero [56]. The models were implemented on a maximum likelihood framework and candidate models were compared using AIC, considering as models with support those with a ΔAIC < 2.0 units from the most parsimonious model [57]. Occupancy analyses were run using the ‘unmarked’ package [58] in R, version 4.1.0 [59].

### Temporal overlap

To test for temporal segregation between jaguarundis and tiger cats at our site, their respective activity patterns were compared, as well as both felid species’ activity patterns in relation to domestic dogs and cats. Initially, timestamps were obtained from the photographic records of each species and transformed into radian times. Afterwards a kernel circular density function was fitted to the data to estimate each species’ activity levels. Diel activity levels among each species pair were compared using a Wald test. To measure the extent of overlap between each species’ diel activity, the coefficient of overlap (Δ_1_) was used; this coefficient ranges from 0 (no temporal overlap) to 1 (complete overlap) and it is used when the sample size for each species is lower than 75 [60], and 95% confidence intervals were obtained from 10,000 smoothed bootstrap samples [61]. Finally, to compare each species activity patterns a randomization test was run using the function *compareCkern()* with 10,000 bootstrap iterations. All analyses were conducted in R, version 4.1.0 [59], using the packages ‘activity’ [62] and ‘overlap’ [63].

## Results

### Spatial co-occurrence

During 8,566 trap-nights, 116 independent records of northern tiger cats, 44 of domestic dogs, 39 of domestic cats, and 21 of jaguarundi were obtained, resulting in a trapping rate per 100 trap nights of 1.35, 0.51, 0.46, and 0.25, respectively.

**Fig 2:**
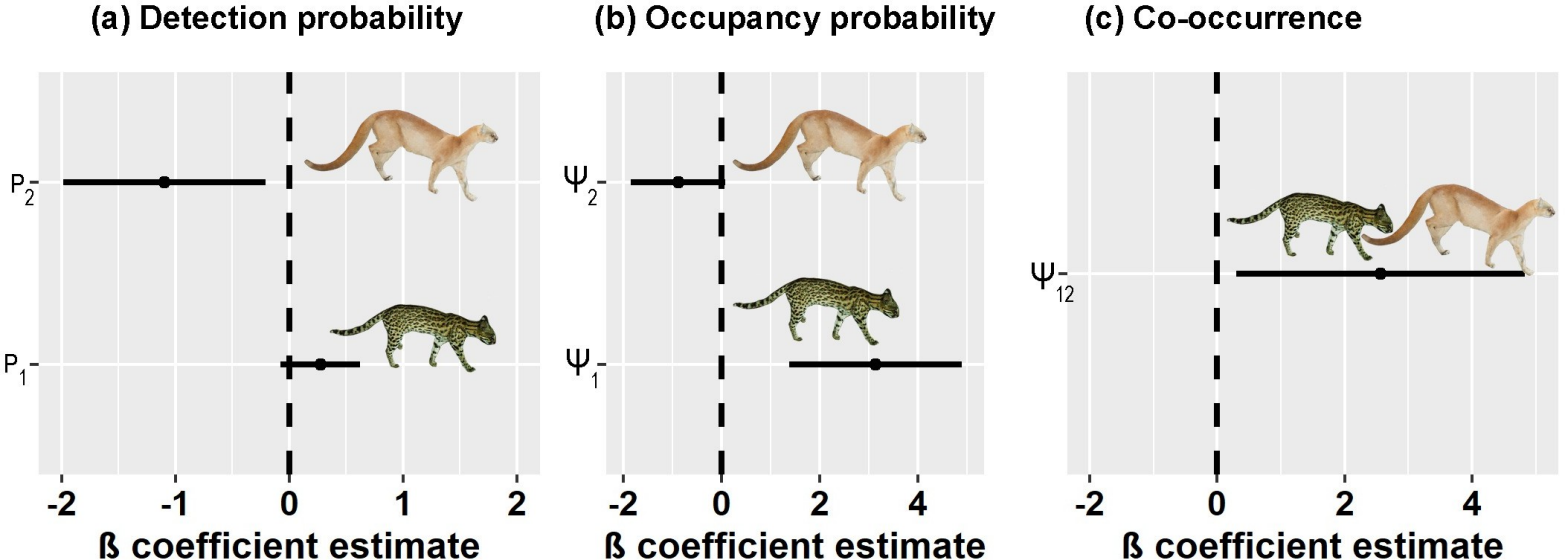
Species responses to covariates. (a) Detection probabilities in response to distance to water sources; (b) Occupancy probabilities in response to Ψ_1_ (percent tree cover), Ψ_2_ (distance to nearest plantations); (c) Pairwise co-occurrence probability.

Evidence of positive co-occurrence was found between both cats, with the independence-assumed model having a ΔAIC of 47.17 (S1 Table). According to the best model, co-occurrence was significantly positive (β = 2.56 ± 1.15; 95% CI: 0.30–4.83). The top five models received almost all the weight, with the top two being 1.80 AIC units apart (Table 2). Detection probability of northern tiger cats was not affected by distance to water bodies, as the confidence interval overlapped with zero (β = 0.27 ± 0.18, 95% CI: -0.08–0.62; Fig 2A). Jaguarundi detectability was higher closer to water bodies (β = -2.25 ± 1.03, 95% CI: -1.98 –-0.21; Fig 2A). Survey effort did not affect the detectability of either species. Mean detection probabilities were 0.10 ± 0.3 for jaguarundis and 0.22 ± 0.04 for northern tiger cats.

**Table 2:** Model selection results showing models with a cumulative AIC weight of 0.95. Ψ represents the probability of habitat use (occupancy parameter), p represents the detection parameter. Species codes are as follow: 1 = northern tiger cat; 2 = jaguarundi; 12 = both. *k* indicates the number of parameters for each model.

Model ^a^	AIC	ΔAIC	AIC_W_	*k*
Ψ_1_(TC) Ψ_2_(AG)Ψ_12_(.)p_1_(WD)p_2_(WD)	558.07	0.00	0.48	9
Ψ_1_(TC) Ψ_2_(AG)Ψ_12_(TC)p_1_(WD)p_2_(WD)	559.88	1.80	0.19	10
Ψ_1_(TC) Ψ_2_(AG)Ψ_12_(WD)p_1_(WD)p_2_(WD)	560.07	2.00	0.18	10
Ψ_1_(TC) Ψ_2_(AG)Ψ_12_(.)p_1_(WD)p_2_(.)	561.72	3.64	0.08	8

^a^ Variables coded as: TC = percent tree cover; AG = distance to plantations; WD = distance to water sources.

**Fig 3:**
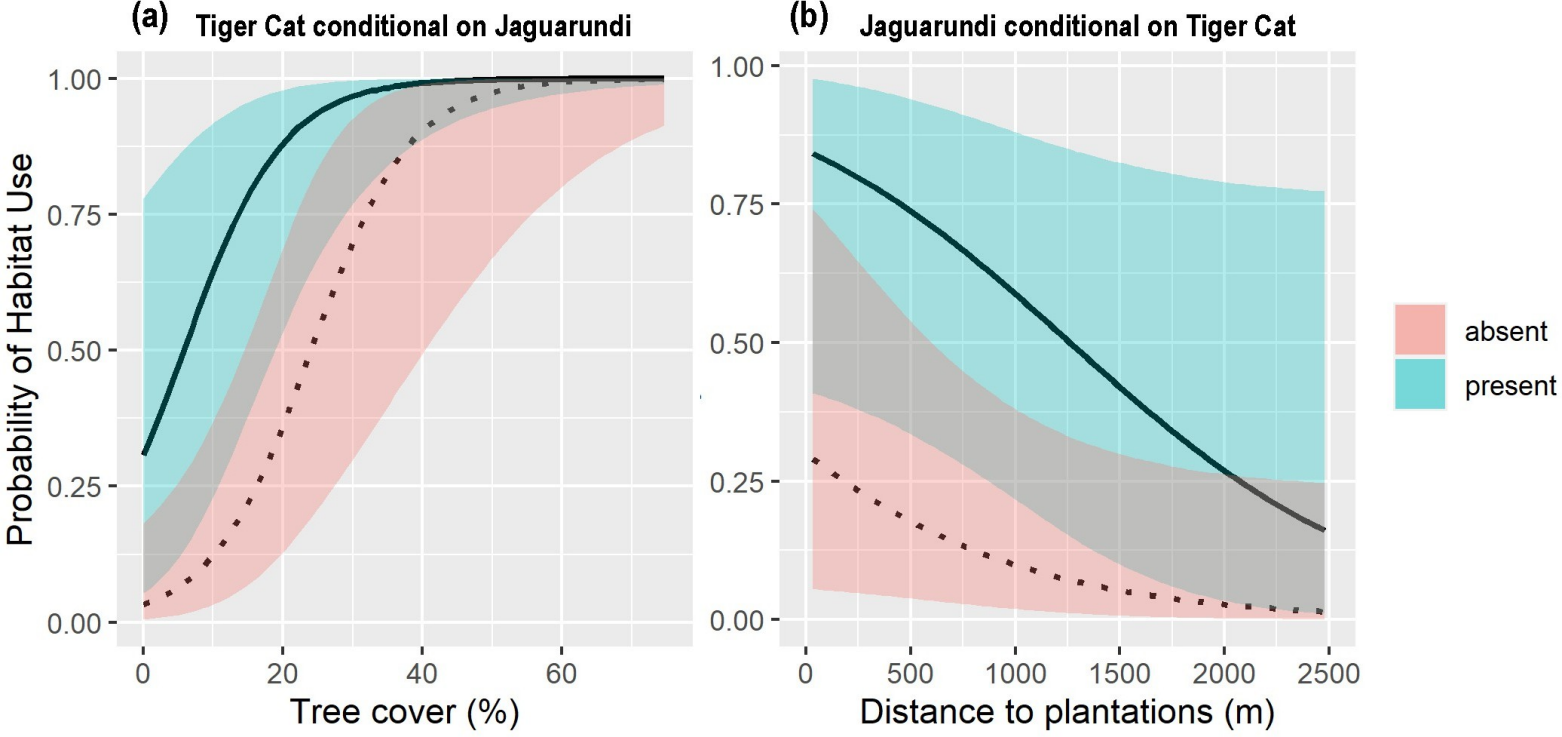
Conditional occupancy probability of (a) Northern tiger cat conditional on jaguarundi and (b) Jaguarundi conditional on northern tiger cat.

Marginal occupancy of tiger cats was higher in areas with denser tree cover (β = 3.14 ± 0.90, 95% CI: 1.37–4.90; Fig 2B). Marginal occupancy of jaguarundi was not significantly affected by distance to the nearest plantation (β = -0.88 ± 0.49, 95% CI: -1.84–0.08; Fig 2B). Northern tiger cats had a higher probability of marginal occupancy (ψ_1_ = 0.62 ± 0.08) than jaguarundis (ψ_2_ = 0.43 ± 0.15). Each species’ respective occupancies were higher in the presence of the other species regardless of covariate values (Fig 3).

### Temporal overlap

**Fig 4:**
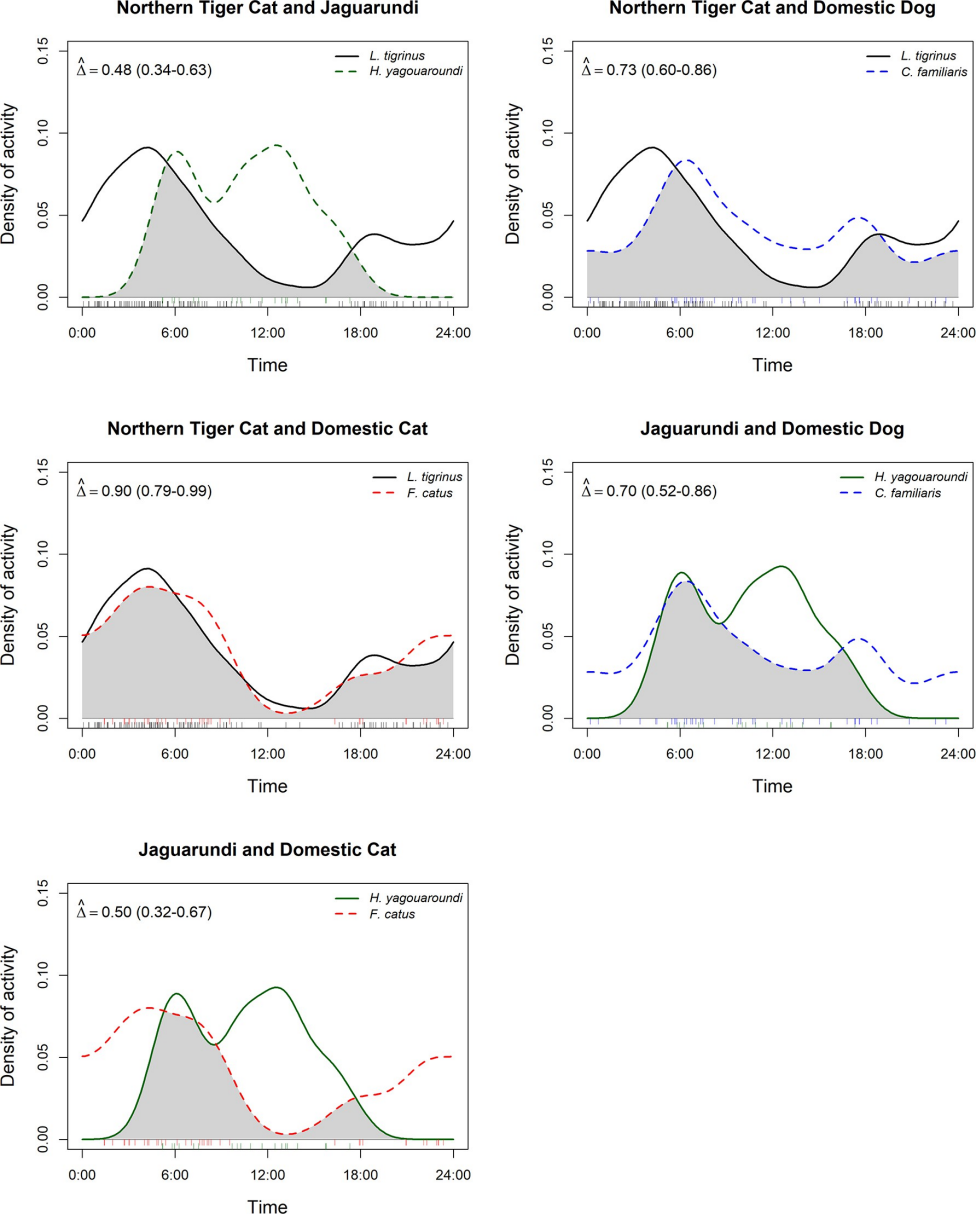
Activity overlap plots among each species pairs.

Evidence of temporal segregation was found between tiger cats and jaguarundis, as well as moderate degree of temporal overlap between jaguarundis and domestic cats, and high temporal overlap between the remaining species pairs (Fig 4). The coefficient of overlap between both wild felid species was 0.48 (95% CI: 0.34–0.63), whereas overlap with domestic dogs was 0.73 for the northern tiger cat (95% CI: 0.60–0.87) and 0.70 for the jaguarundi (95% CI: 0.52–0.86). Overlap was greatest between northern tiger cats and domestic cats at 0.90 (95% CI: 0.79–0.99), whereas overlap between domestic cats and jaguarundis was 0.50 (95% CI: 0.32–0.67). Activity levels were similar across all species pairs (Table 3). There were significant differences in the distribution of diel activity patterns among all species pairs except for northern tiger cat–domestic cat, and jaguarundi–domestic dog (Table 3). Peak activity for jaguarundi was after sunrise and midday, with no records after 18:00. Tiger cat activity was mostly nocturnal, with an activity peak just before sunrise.

**Table 3:** Pairwise comparison of species’ diel activity levels and patterns at the Tamanduá Ranch.

Species pair	Wald test		Randomization test		
	W	*P*	Observed overlap	Null distribution	*P*
Tiger cat–jaguarundi	0.01	0.94	0.48	0.78	<0.01*
Tiger cat–domestic dog	0.12	0.73	0.74	0.84	0.04*
Tiger cat–domestic cat	0.50	0.48	0.90	0.84	0.87
Jaguarundi–domestic dog	0.13	0.72	0.70	0.77	0.18
Jaguarundi–domestic cat	0.44	0.51	0.50	0.76	<0.01*

* Denotes statistical difference using a significance level of 0.05.

## Discussion

Evidence was found of positive co-occurrence among jaguarundis and northern tiger cats. Hence, there does not seem to be spatial segregation between both species at our study site. A lack of spatial avoidance between these felids has been detected at other sites in the Caatinga and Cerrado/Atlantic Forest/pasture mosaic elsewhere in Brazil [11]. Had there been any form of spatial independence between both species, one would have expected the best model to include the *f*_12_ parameter set as zero. Instead, evidence was found of temporal segregation, with low activity overlap and significant differences in activity patterns between both felid species.

Prey availability could be a major factor shaping the co-occurrence of both species. Despite differences in size, both cats tend to feed on small mammals (<1 kg of body weight). Dietary studies comparing both species are non-existent, but mean weight of mammalian prey between jaguarundis and southern tiger cats (*L*. *guttulus*), an ecological-equivalent of the northern tiger cat from the Atlantic Forest, differed significantly at several sites in which they occurred in sympatry [10,64,65]. At the same time, dietary overlap between jaguarundis, southern tiger cats, and ocelots could be high in areas of the Atlantic Forest [66]. Elsewhere, dietary segregation allows carnivores of similar size and ecological niche to coexist in areas with a high prey base [67]. Nevertheless, carnivores often employ other strategies to avoid competition. For instance, in a prey-rich area of the Nepalese Terai, Bengal tigers (*Panthera tigris*) and leopards (*Panthera pardus*) did not dietarily segregate, but rather adopted spatiotemporal segregation strategies [68]. At the Tamanduá Ranch, it could be possible that the positive co-occurrence observed between both felid species reflects the fact that they are sharing the habitats with the greatest abundance of prey, with their temporal segregation facilitating coexistence. Nevertheless, each species’ respective marginal occupancy and detection probability varied along different covariates, which suggests that they also have different environmental preferences.

Northern tiger cat marginal occupancy increased with higher tree cover, which is a pattern that is in agreement with previous research in the Caatinga biome. At several sites in Rio Grande do Norte state, for example, northern tiger cats had a higher probability of occurrence in areas of greater forest cover [33]. As a species, tiger cats are generally associated with tree cover, particularly in savanna and semi-arid environments. The highest densities documented for the species come from the woodland and shrubland savannas of Mirador State Park, with the species being absent from the open savanna formations of the this park [23]. At our site, the species preferred denser areas over the open Caatinga vegetation. From a management perspective, this is important, as it suggests that for private lands to better protect the species, dense formations should be preserved. Jaguarundis did not respond to different levels of tree cover, which suggests that the species is less constrained to different vegetation types than the northern tiger cat. Throughout their range, jaguarundis have been known to use a wide variety of habitats, including savannas, thorn shrub and dense forests [18,36,69–71].

Despite our predictions, distance to water sources did not influence habitat use by the small felids. Nevertheless, the variable did significantly affect the detection probability of jaguarundis. Across the Neotropics, jaguarundis have been thought to use areas closer to water bodies based on both home range and habitat use analyses [72–74]. This apparent preference for water sources has been suggested to reflect usage of riparian habitats [28]. Riparian vegetation with some cover does occur at some of the lakes in the property, and this type of vegetation provides shelter and likely a prey base for wild cats [75]. In other dry and semi-arid environments, species richness and abundance of sigmodontine rodents were higher in riparian habitats and gallery forests [76,77]. Thus, it is possible that prey availability is driving detectability of jaguarundis at our site. Nevertheless, tiger cats, which would also benefit from increased prey availability, did not exhibit this pattern. Elsewhere in the Caatinga and other non-forested environments of northern Brazil, northern tiger cat occupancy or detection probabilities were not affected by distance to water sources [33,78]. Since, in the semi-arid Caatinga, water accumulates in pools on rocky formations during the rainy season, it is believed unlikely that man-made lakes are a major source of water for our study species. The extreme temperatures of the Caatinga coupled with diurnal habits could result in jaguarundis selecting areas closer to water sources as heat refuges, which has been documented in several vertebrate species [79]. Further research is needed to discern the relationship between jaguarundi habitat use patterns and permanent water sources.

Our analysis suggests that the major segregation mechanism between tiger cats and jaguarundis at our site is temporal partitioning, with low activity overlap between the two species and a significant difference in their activity patterns. Tiger cats were mostly active at night and jaguarundis exclusively at daytime. Jaguarundis are classified as diurnal animals [27]. A review of 10 published and unpublished works [28] found no records for jaguarundis between 0:00–4:00 am and only one record between 20:00–23:59. At our site, only one jaguarundi record came from the twilight hours (one hour before/after sunrise/sunset respectively), with the rest being during the daytime (93.75% diurnal). Throughout the Neotropics, jaguarundi show low activity overlap with sympatric felids [17,78,80]. Northern tiger cats, though often described as nocturnal, do exhibit considerable daytime activity in some areas [15]. At our study site, 29.7% of northern tiger cat records were during daytime which is considerably higher than in the Caatinga of Rio Grande do Norte, where diurnal activity was 12.7% [37]. Nevertheless, the species was predominantly nocturnal at our site, thus facilitating coexistence with the jaguarundi. This pattern seems to be consistent regardless of the presence of dominant competitors. At a large Caatinga remnant in the Lajes municipality (Northeast Brazil), where ocelots are known to occur, northern tiger cats and jaguarundis also exhibited low temporal overlap and significantly different activity patterns [81].

The presence of domestic dogs and cats in private lands of the Caatinga could pose a major threat for long-term small felid conservation. At the Tamanduá Ranch, domestic dogs exhibited high temporal overlap with both tiger cats and jaguarundis. Although the randomization test suggests different activity patterns between tiger cats and domestic dogs (Table 3), temporal overlap between both species was still high. The difference lies in the timing of activity peaks. While northern tiger cat activity peaked between midnight and sunrise (Fig 4), domestic dog activity peaked just after sunrise. In spite of this difference in peak activity, temporal overlap between northern tiger cats and dogs in our study site was higher than in Mirador State Park, in the northern Cerrado [82]. Further research should address if this difference in activity peaks is a result of northern tiger cats avoiding domestic dogs temporally. High temporal overlap between domestic dogs and native mesopredators, including our two study species, has already been documented in other areas with no evidence of temporal segregation [38]. Predation by domestic dogs could be a potential threat for our study species; however, in all likelihood, disease transmission would be the main threat in the long term as it is for northern tiger cats at Mirador State Park [23]. At our study site, the number of records of domestic dogs and cats was considerably higher than the number of records of jaguarundis, but lower than the number of records of tiger cats. Domestic dogs were detected at 67% of camera traps that recorded jaguarundis and 59% of camera traps that recorded northern tiger cats. Domestic cats were detected at 55% of camera traps that recorded jaguarundis and 35% of camera traps that recorded northern tiger cats. With regards to domestic cats, the main threat comes in the form of disease transmission since they are not capable of killing an adult wild cat. Due to our limited sample size, it was not possible to model domestic dog occupancy along with that of the two cats in a three-species model. In some areas, free ranging domestic dogs influence the habitat selection patterns of wild mesopredators [83]. Thus, testing for spatial interactions between domestic dogs and wild felids is something that should be done in future works.

### Implications for small felid conservation and future directions

Herein, an assessment is provided of spatiotemporal mechanisms of coexistence between two sympatric felids in the semi-arid Caatinga; whose conservation is highly dependent on private lands. Our results suggest that both species share the same landscape, and that temporal segregation is a major mechanism that allows for their coexistence in the same area. This lack of spatial avoidance is important because it suggests that conservation strategies can target both species at once and within the same area, provided enough habitat and connectivity with other habitat patches are available. Both species have large home ranges given their body size, reaching up to 94 km^2^ for jaguarundis and up to 17 km^2^ for northern tiger cat [9,74]. Sympatric carnivores may leverage the dietary niche dimension to avoid competition; hence, future works should explore possible dietary segregation among small felids in these privately owned areas.

The small Neotropical felid guild’s long-term conservation is dependent upon private lands outside the major reserves where ocelots are highly abundant [10]. This means that further research exploring co-occurrence patterns between the smaller felids in private lands in other biomes should be conducted. Furthermore, it is important to take into account the presence of other carnivores. At the Tamanduá Ranch we have a small carnivoran guild in which the jaguarundi and northern tiger cat are the only hypercarnivores. The area is also home to crab-eating foxes (*Cerdocyon thous*) and crab-eating raccoons (*Procyon cancrivorus*), but neither of the two is likely to compete with the cats due to different dietary preferences. Nevertheless, at other private reserves, small felids are likely to share the environment with larger carnivores such as the ocelot, or even the coyote (*Canis latrans*) in Central America. Interactions with these species should be modeled whenever possible in order to obtain better knowledge of small felid habitat preferences in privately-owned areas. A study carried out in the Private Natural Heritage Reserve Serra das Almas, in the Brazilian state of Ceará, a reserve with twice the area of Fazenda Ranch, indicated that tiger cats and jaguarundis are very rare, most likely due to the locally-abundant ocelot [84]. Interestingly, in Boqueirão da Onça National Park, in the Caatinga of Bahia, ocelots, which were highly abundant, had a high temporal overlap with tiger cats, but had no effect on habitat use by jaguarundis. There, tiger cats were restricted to areas of shrub Caatinga, whereas the ocelot used habitats with denser vegetation. [78]. In this area, neither jaguar, nor puma, caused any effect on the small cats [78]. Further studies are needed in which the full cat assemblage is present.

Besides co-occurrence patterns, assessing long-term persistence of small felids in such private landscapes is important. Our analysis just provides a snapshot of the environmental drivers of habitat use for both species while also considering their interspecific interactions. Nevertheless, long-term data would be needed in order to see whether the animals persist in these private lands. At the Tamanduá Ranch, occurrence probability of tiger cats and jaguarundis did not change between 2010–2014 [32]; nevertheless, the density of both species declined between 2011 and 2019, which would represent a considerable population drop over a period of three generations [85].

From a management perspective, the main issue to focus on at our site would be the presence and number of domestic dogs in the area. Relative abundance of domestic dogs was more than twice of that of jaguarundis at the Tamanduá Ranch; however, conversely it was lower than that of northern tiger cats. This pattern contrasts with what has been found in several areas of the Atlantic Forest, where domestic dogs outnumbered all the carnivoran species sampled [86]. The dogs recorded in our cameras are either feral or belong to people living outside the reserve. Addressing this issue will require different management approaches, such as vaccination campaigns and environmental education, in order to motivate owners to not allow their dogs to roam freely.

In conclusion, research on habitat use and co-occurrence patterns of small felids in private lands is important for proper conservation and management actions. Our results suggest that in a scenario of high human pressure (livestock raising, agriculture, and proximity of households), albeit with no poaching and good amount of natural vegetation left, northern tiger cats and jaguarundis are able to share the same habitat and coexist through temporal segregation. Nevertheless, important attention must be paid to the potential threat from domestic dogs, since privately owned areas in the Neotropics often have these animals present. Depending on the anthropogenic activities practiced, issues such as human-wildlife conflict and rodenticide use can be threats as well. Notwithstanding, our results suggest that conservation actions can target small felids together in these private lands, thus improving their chances for long-term survival.

## Supporting information

S1 Table Full model selection table. Ψ represents the probability of habitat use (occupancy parameter), p represents the detection parameter. Species codes are as follow: 1 = northern tiger cat; 2 = jaguarundi; 12 = both. *k* indicates the number of parameters for each model.
(DOCX)

S1 Dataset Raw data used for running the analyses of this manuscript. (XLSX)
