# Supplementary material for: Interspecific patterns of small cats in an intraguild-killer free area of the threatened Caatinga drylands, Brazil
Source: PLoS One. 2023 Apr 21;18(4):e0284850. doi: 10.1371/journal.pone.0284850 (PMC10121031; doi:10.1371/journal.pone.0284850)
Supplement: S1 Table — Ψ represents the probability of habitat use (occupancy parameter), p represents the detection parameter. Species codes are as follow: 1 = northern tiger cat; 2 = jaguarundi; 12 = both. k indicates the number of parameters for each model. (DOCX) [file pone.0284850.s001.docx]

S1 Table. Full model selection table. Ψ represents the probability of habitat use (occupancy parameter), p represents the detection parameter. Species codes are as follow: 1 = northern tiger cat; 2 = jaguarundi; 12 = both. *k* indicates the number of parameters for each model.

| **Model ^a^** | **AIC** | **ΔAIC** | **AIC_W_** | ***k*** |
| --- | --- | --- | --- | --- |
| Ψ_1_(TC) Ψ_2_(AG)Ψ_12_(.)p_1_(WD)p_2_(WD) | 558.07 | 0.00 | 0.46 | 9 |
| Ψ_1_(TC) Ψ_2_(AG)Ψ_12_(TC)p_1_(WD)p_2_(WD) | 559.88 | 1.80 | 0.19 | 10 |
| Ψ_1_(TC) Ψ_2_(AG)Ψ_12_(WD)p_1_(WD)p_2_(WD) | 560.07 | 2.00 | 0.17 | 10 |
| Ψ_1_(TC) Ψ_2_(AG)Ψ_12_(.)p_1_(WD)p_2_(.) | 561.72 | 3.64 | 0.075 | 8 |
| Ψ_1_(TC) Ψ_2_(AG)Ψ_12_(.)p_1_(WD)p_2_(effort) | 561.84 | 3.76 | 0.07 | 8 |
| Ψ_1_(TC) Ψ_2_(WD)Ψ_12_(.)p_1_(WD)p_2_(.) | 565.20 | 7.13 | 0.013 | 8 |
| Ψ_1_(TC) Ψ_2_(TC)Ψ_12_(.)p_1_(WD)p_2_(.) | 565.92 | 7.85 | 0.01 | 8 |
| Ψ_1_(TC) Ψ_2_(.)Ψ_12_(.)p_1_(WD)p_2_(.) | 566.42 | 8.34 | 0.00 | 7 |
| Ψ_1_(TC) Ψ_2_(.)Ψ_12_(.)p_1_(.)p_2_(.) | 567.27 | 9.19 | 0.00 | 6 |
| Ψ_1_(TC) Ψ_2_(.)Ψ_12_(.)p_1_(effort)p_2_(.) | 567.40 | 9.33 | 0.00 | 7 |
| Ψ_1_(WD) Ψ_2_(.)Ψ_12_(.)p_1_(.)p_2_(.) | 596.80 | 38.72 | 0.00 | 6 |
| Ψ_1_(.) Ψ_2_(.)Ψ_12_(.)p_1_(.)p_2_(.) | 604.00 | 45.92 | 0.00 | 5 |
| Ψ_1_(.) Ψ_2_(.)Ψ_12_(.)p_1_(.)p_2_(0) | 605.24 | 47.17 | 0.00 | 4 |
| Ψ_1_(AG) Ψ_2_(.)Ψ_12_(.)p_1_(.)p_2_(.) | 605.95 | 47.88 | 0.00 | 6 |
| Ψ_1_(Y) Ψ_2_(.)Ψ_12_(.)p_1_(.)p_2_(.) | 607.80 | 49.73 | 0.00 | 8 |
| Ψ_1_(.) Ψ_2_(Y)Ψ_12_(.)p_1_(WD)p_2_(.) | 609.30 | 51.22 | 0.00 | 8 |

^a^ Variables coded as: TC = percent tree cover; AG = distance to plantations; WD = distance to water sources.
